# De hegemonías, subalternidades y posibles contrahegemonías: El caso de la medicina tradicional de los pueblos indígenas de México

**DOI:** 10.18294/sc.2024.4843

**Published:** 2024-06-10

**Authors:** Eduardo L. Menéndez

**Affiliations:** 1 Doctor en Ciencias Antropológicas. Doctor Honoris Causa por la Universitat Rovira i Virgili, Catalunia; Universidad Nacional de Rosario, Argentina; y Universidad Nacional de Lanús, Argentina. Profesor-investigador emérito, Centro de Investigaciones y Estudios Superiores en Antropología Social (CIESAS), México DF, México. emenendez1@yahoo.com.mx Universidad Nacional de Lanús Universidad Nacional de Lanús Argentina; Universitat Rovira i Virgili Catalunia España; Universidad Nacional de Rosario Argentina

**Keywords:** Medicina Tradicional Indígena de las Américas, Medicina Clínica, México, Traditional Indigenous Medicine of the Americas, Clinical Medicine, Mexico

## Abstract

Toda una serie de procesos conducen a la disminución del uso de la medicina tradicional por los pueblos indígenas de México, incluyendo la reducción del número de curadores tradicionales y la expansión directa e indirecta de la biomedicina. En este ensayo se aborda el papel nuclear que tienen estos procesos en las relaciones de hegemonía/subaltenidad que se dan en los diferentes campos de la realidad y, especialmente, en los procesos de salud-enfermedad-atención-prevención, dado que no se generan procesos contrahegemónicos o, los que surgen, han sido ineficaces para enfrentar la hegemonía social en general y biomédica en particular.

## Abstract (en)

A whole series of processes lead to the decrease in the use of traditional medicine by the indigenous peoples of Mexico, including the reduction in the number of traditional healers and the direct and indirect expansion of biomedicine. This essay addresses the central role these processes play in the relations of hegemony/subalternity that occur in different fields of reality, and especially in the health-illness-care-prevention processes, given that counter-hegemonic processes are not generated, or those that do arise have been ineffective in confronting social hegemony in general and biomedical hegemony in particular.

## INTRODUCCIÓN

La bibliografía analizada, las conversaciones con algunos de los principales especialistas en medicina tradicional, así como mis propios estudios, indican que la medicina tradicional sigue siendo la más usada por los pueblos originarios de México, pero con tendencias a disminuir sus usos en las generaciones más jóvenes; dado que en estas se incrementan los usos de la biomedicina, y se da una articulación en el uso de ambas sanaciones. Más aún, disminuye cada vez más el número de curadores tradicionales, se da en una parte de estos un proceso de biomedicalización, así como se registra la desaparición de algunas enfermedades tradicionales y su reemplazo o coexistencia con enfermedades alopáticas^1^^,^^2^^,^^3^^,^^4^.

En este proceso operan varios factores que tienen que ver no solo con la expansión biomédica, sino también con la expansión en forma directa e indirecta de la sociedad dominante en los diversos campos de las personas indígenas, especialmente, en los campos de la ocupación, educación, religión, tecnologías de comunicación e información, manejo de lenguaje, etc., que inciden en los usos y costumbres de los pueblos originarios. Y ello no solo en el ejercicio de saberes (representaciones y prácticas) específicos, sino también a través de las relaciones de hegemonía/subalternidad/contrahegemonía (H/S/C) a nivel general y de ambas formas sanadoras en particular, señalando que tanto a nivel general como especifico dichos procesos de H/S/C necesitan ser observados en su relación con los procesos de poder, de explotación económica, de violencias que se ejercen sobre los sectores subalternos. Por eso, en este texto presentaré información en términos sintéticos sobre el papel de la biomedicina y de la medicina tradicional en los procesos de H/S/C, pero acompañados de información sociocultural y económico-política, que no tienen que ver en forma directa con los procesos de salud-enfermedad-atención-prevención, pero que son parte de un conjunto de procesos a través de los cuales se generan relaciones de H/S/C potenciándose mutuamente.

## LA CONSTITUCIÓN DE LOS PROCESOS DE HEGEMONÍA/SUBALTERNIDAD/CONTRAHEGEMONÍA

Como sabemos, las relaciones de H/S/C se vienen constituyendo desde principios del siglo XVI entre los pueblos mesoamericanos y los conquistadores/colonizadores, junto con el derrumbe demográfico, la dominación política, la evangelización cristiana y la explotación económica. Entre los siglos XVI y XIX, se generó un proceso de inferiorización, de exclusión y de racialización de la población nativa, y más tarde de la población mestiza, que contribuyó a subalternizarlas en todos los campos de la realidad. Más aun, como mecanismo de dominación, los conquistadores establecieron un proceso de homogenización que convierte en “indios” a todos los pueblos originarios concebidos como un bloque demográfico y cultural que experimenta el derrumbe demográfico en todos sus sectores sociales.

Si reitero lo del derrumbe demográfico es porque entre principios del siglo XVI y parte del siglo XVIII -y debido a las epidemias de varias enfermedades infectocontagiosas-, se calcula que despareció el 90% de la población mesoamericana, quedando reducida en 1620 a 730.000 habitantes, lo que constituye un 3% de la población original^5^. Este es uno de los procesos que más posibilitó la unificación de los indígenas en un solo bloque, según la mirada de los colonizadores, pero también de los colonizados, dado que en todas partes experimentaban que mientras los nativos morían masivamente debido a las nuevas enfermedades infectocontagiosas como la viruela, el sarampión, la tos ferina, etc., traídas por los europeos, lo mismo no ocurría con los colonizadores, lo que constituía un importante mecanismo de hegemonía/subalternidad basada en la mortalidad diferencial. Este proceso de mortalidad diferencial duró por lo menos tres siglos, y se dio en pueblos mesoamericanos e hispanos que, cuando se “encontraron” tenían una esperanza de vida similar, o incluso los mesoamericanos tenían una esperanza mayor, ya que sería de unos 35 años, respecto de los españoles que era de unos 30 años.

Si bien la corona española impulsó intencionalmente un constante proceso de evangelización, necesitamos asumir que la asimilación, aculturación, hibridación o como se la quiera llamar, se debió estructuralmente al funcionamiento social, económico y cultural que los conquistadores impusieron en la vida cotidiana indígena, en la medida que entraron en relación directa e indirecta con ellos. Es decir, no solo surge a partir de una intencionalidad asimiladora, sino que el funcionamiento en la vida cotidiana va normalizando no conscientemente dicho proceso. Cabe aclarar que la aculturación/hibridación supone un proceso en el que los subalternizados dan un significado y sentido propio a los nuevos usos, pero dentro de los márgenes que establecen los colonizadores^5^. Más aún, necesitamos observar si desde principios del siglo XVI hasta la década de 1970, los pueblos originarios fueron siempre subalternos o generaron no solo una cultura propia, sino una ideología que les posibilitara transaccionar favorablemente en forma directa y/o indirecta con la cultura/ideología de los colonizadores, y más tarde con los sectores sociales dominantes durante la etapa independiente. Personalmente reconozco tres formas principales de entender lo ideológico: a) como concientización y movilización; b) como justificación y c) como falsa conciencia. De estas tres formas, utilizo sobre todo la primera posibilidad, que sostiene que la cultura solo opera como reproductora de los sistemas sociales -lo práctico inerte que proponía Sartre-, por lo que se necesita generar ideología para impulsar cambios socioculturales en el sentido y orientación que los actores sociales buscan establecer. Esta concepción puede incluir lo ideológico como mistificación y/o falsa conciencia según los objetivos buscados. Y todo ello pensado como marco referencial, porque lo que me interesa es observar la trayectoria social e ideológica de esa relación, para ver si ha incidido en la emergencia de proyectos y sobre todo de acciones contrahegemónicas propias, generadas por los pueblos originarios desde la década de 1970 hasta la actualidad.

Mientras los colonizadores impulsaron toda una serie de saberes culturales, sociales y económicos que pasaron a ser parte de la vida cotidiana de la población mesoamericana, no observamos que durante el periodo colonial y el periodo independiente los grupos indígenas hayan generado concepciones ideológicas contraculturales que enfrentaran los saberes dominantes y los desplazaran o por lo menos convivieran en forma simétrica e igualitaria, lo que no niega que los pueblos originarios -sobre todo los más aislados- desarrollaran un campo sociocultural relativamente autónomo. Pero tanto las propuestas capitalistas liberales y conservadoras como los cuestionamientos ideológicos y políticos anticapitalistas desarrollados por corrientes teóricas y políticas de izquierda desde mediados del siglo XIX no incluyeron la cuestión indígena. Y menos aún plantearon que la vía indígena constituyera una alternativa a la vía capitalista. Por supuesto que reconocemos la existencia de enfrentamientos en el caso latinoamericano, como fue el caso paradigmático de Tupac Amaru en el área andina, así como procesos de resistencia en gran medida basados en la resignificación que los nativos hicieron con los usos impuestos por los colonizadores, pero lo que pasó con estos procesos, no alteró en términos contrahegemónicos las relaciones de hegemonía/subalternidad. Los procesos políticos de enfrentamiento al poder español fueron desarrollados básicamente por el sector criollo, sin que los indígenas tuvieran que ver con ello, sino como fuerza de lucha. Mas aun, el pensamiento europeo desarrolló durante el siglo XIX teorías evolucionistas, difusionistas y racistas que legitimaron el dominio y la hegemonía europea, así como la inferioridad y la subalternidad de los indígenas, lo cual permeó no solo las concepciones liberales y conservadoras sino también las propuestas teóricas e ideológicas progresistas^6^. De tal manera que durante el siglo XIX las propuestas sociales progresistas se basaron en concepciones anarquistas, socialistas y más tarde comunistas, que planteaban la necesaria caída del capitalismo, pero sin que en sus propuestas apareciera el actor indígena, sino que la posibilidad de lucha y cambio se depositaron en el proletariado y en el campesinado que incluyen -sobre todo el campesinado- a la población nativa, pero en términos de campesinado y no de indígenas; y esto lo observamos paradigmáticamente en el caso de la revolución mexicana. Ello no niega que dentro del pensamiento europeo entre los siglos XVI y XIX se generaron concepciones que defendían a los pueblos originarios y cuestionaban la colonización^7^, pero en ningún momento fueron hegemónicas.

Esta trayectoria, en la que está ausente el indígena, sigue hasta la década de 1970, cuando comienza a surgir el actor nativo como posibilidad política, que emerge, en 1994 con el Ejercito Zapatista de Liberación Nacional (EZLN), quien constituye hasta ahora la expresión máxima de este proceso a nivel de México, aunque con características que algunos analistas cuestionan, ya que lo observan como un movimiento no indígena, por lo menos en su organización y surgimiento, ya que fue desarrollado desde perspectivas marxistas por sujetos no indígenas.

De tal manera que, a nivel de México, y de varios países latinoamericanos, uno de los objetivos de los sectores sociales e intelectuales progresistas es la caída del capitalismo y su reemplazo. Y esta posibilidad se agudizó en varios momentos del siglo XX, lo que volvió a ocurrir en el siglo XXI con la crisis económica de 2007-2008 y con la pandemia de covid-19 en 2020-2022, durante las cuales parecía que la caída del capitalismo estaba a la vuelta de la esquina como surge de las propuestas de Slavoj Žižek, Raúl Zibechi y compañía, cuando lo que estábamos viviendo era que el conjunto de las sociedades se habían vuelto capitalistas. Esto no niega que en algún momento el capitalismo sea reemplazado. Pero no por el BRICS, o por la Rusia de Putin o por la China pos Mao Tse Tung, o por el Vietnam pos Ho Chi Minh dado que las mismas constituyen formaciones capitalistas.

La situación colonial y el colonialismo “interno” mantuvieron a los pueblos originarios en una situación de subalternidad, y de aparente marginalidad; y digo aparente porque dichos pueblos constituyeron -y siguen constituyendo- entre otras cosas el principal reservorio de mano de obra barata, que propició el desarrollo capitalista de México. Si bien la mayoría de antropólogos y antropólogas en las décadas de 1980, 1990 y principios de 2000 no analizaron a los indígenas como clase social, sino como comunidades culturales, sin embargo -y lo subrayo- el proceso permanente de migración rural/urbana va a convertir a los indígenas en estrato social urbano y, a una parte, en proletariado. Como dice Warman:

Alguien inventó el mito de la explotación colonial como opuesta a la explotación clasista [pero la] explotación colonial y la explotación salarial o capitalista son modalidades de la explotación clasista. La realidad de los países coloniales sugiere que estas variedades no son contradictorias […] sino líneas complementarias que maneja en su beneficio un solo grupo en el poder.^8^

Y uno de los que inventaron lo señalado por Warman fue un antiguo compañero suyo; es decir Guillermo Bonfil Batalla, quien considera que no es correcto analizar la población indígena en términos de clases, sino que debe ser analizada en términos de situación colonial entendida como aquella “…en la que un grupo con cultura propia similar somete, se superpone, domina y explota a otros que poseen una cultura diferente”^9^. Y Bonfil subraya lo de cultura diferente, sosteniendo que los pueblos prehispánicos cuando fueron invadidos por los conquistadores se

…volvieron sobre sí mismos y reforzaron los nexos internos de la comunidad: esto favoreció a los sistemas sociales que hoy llamamos corporales o corporativos […] La identidad étnica se apoya en estos casos, en la pervivencia de una cultura sometida de carácter marcadamente defensiva y aislante.^9^

El análisis de Bonfil es ahistórico, pues deja de lado varios aspectos y procesos que cuestionan su propuesta, y de los cuales solo señalamos dos: a) de sus propuestas surge que durante cinco siglos no se habría cambiado el núcleo cultural de los pueblos originarios; y b) ignora la existencia de un constante proceso migratorio, que se agudiza en ciertos momentos, y que convertirán a México en una sociedad urbana gracias, en gran medida, a que los indígenas se convirtieron cada vez más en mestizos urbanos. Este proceso no solo opera en México, sino en el conjunto de los países latinoamericanos:

En los valles y las montañas de Bolivia, Perú, Ecuador, Guatemala y México, las largas historias de interacción con el Estado colonial y los Estados nacionales han erosionado las identidades originales, borrando las diferencias entre los grupos autóctonos y dando lugar a lo que podría describirse como identidad genérica de adscripción indígena. Con el tiempo, los grupos indígenas fueron desarrollando un sentido de identificación local, por lo cual su percepción de sí mismos como tales se arraigaba cada vez más en su pertenencia a una u otra comunidad específica. Paralelamente, el mestizaje y las políticas de modernización y homogeneización cultural fueron ahondando la disolución del referente indígena en los sectores urbanos y en algunos casos rurales.^10^

Pero, además, los análisis tipo Bonfil no solo son ahistóricos, sino que no analizan la realidad como proceso; pues no cabe duda de que los pueblos originarios tienen fuertes diferencias con la sociedad dominante; pero pese a ello se van a convertir, como ya señalé, en el reservorio de mano de obra barata para el desarrollo capitalista mexicano, durante toda la trayectoria colonial e independiente. Más aun, este proceso está generando que cada vez más indígenas residan en el medio urbano. Así observamos que, en 2014, el 52% de los indígenas brasileños vivían en medios urbanos; en Bolivia, el 53%; en Chile, el 64% y, en México, el 36%. Cabe subrayar además que, en México, en la última década, más del 75% de los indígenas ya no hablaban su lengua, es decir, solo hablaban la lengua del “otro”, lo que no solo hegemoniza, sino que excluye la lengua nativa.

Lo que necesitamos asumir es que toda una serie de procesos aseguran, por lo menos durante un tiempo, la dominación y hegemonía de los sectores dominantes. Y de esos procesos voy a señalar tres de los que considero básicos en térmicos estructurales. En primer lugar, observamos que la aculturación/asimilación/hibridación opera funcionalmente en la vida cotidiana de los diversos sectores sociales; es en ese funcionamiento que se reproduce la dominación y la estructura de clases y la inferiorización étnica. En segundo lugar, que los sujetos reproducimos en la vida cotidiana a la sociedad dominante, aun cuando una parte de sus miembros la cuestionen en términos no solo discursivos sino de acciones. Y, tercero, que los sectores dominantes resignifican a los sectores subalternos atribuyéndoles características que legitiman su dominación, y que los propios grupos dominados hacen suya, por lo menos durante un tiempo, como encontré en mi estudio de la migración europea a una comunidad rural de la provincia argentina de Entre Ríos, en la cual observé que los sectores dominantes lo constituían, en la década de 1960, población italiana y española y sus descendientes, mientras los sectores subalternos estaban constituidos por la población nativa. Pero ocurría que gran parte de la población “nativa” era española o descendientes de población española del pueblo Algarrobo de Andalucía, quienes constituían gran parte de los peones rurales. Y así, los sectores dominantes -constituidos por población española y descendientes de pobladores de las zonas del norte de España (Asturias, Cantabria y País Vasco)- habían convertido en nativos argentinos a españoles del sur de la península, atribuyéndoles las características idiosincráticas de los nativos: holgazanes, no les gusta trabajar, se gastan de inmediato lo que ganan, fuerte alcoholización, no mejoran su situación social por sus incapacidades, etc.^11^.

Por lo tanto, una de las tareas básicas de la dominación, y sobre todo de la hegemonía, fue construir un “otro” caracterizado por aspectos negativos que justifican su dominación, siendo el principal aspecto el racismo, que implica no solo la inferioridad del otro, sino la diferenciación radical, dado que remite a aspectos biológicos e incluso genéticos. Pero en el caso de México, oficialmente el racismo no existe; más aún se lo cuestiona tanto respecto de la población indígena, como de la población de origen africano. Pero el racismo es parte de la vida cotidiana mexicana referida no solo a población indígena y africana, sino también a población mestiza. Más aun, mientras la población mestiza es racializada por los sectores medios y altos blancos, la población mestiza a su vez racializa a la población indígena^12^^,^^13^^,^^14^. El racismo antiindígena fue reconocido en México por la Antropología desde, por lo menos, la década de 1950, siendo ejercido no solo por los blancos, sino especialmente por los mestizos^15^. Dicho racismo constituye el principal mecanismo ideológico que se genera en la situación colonial, persistiendo hasta la actualidad^16^^,^^17^^,^^18^. Aclaremos que el racismo generado en el siglo XIX pretende basarse en concepciones científicas, que establecen al racializado no solo como inferior, sino como radicalmente diferente, y por lo tanto sin potencialidad para integrarse a la sociedad dominante. Y si bien esto constituye un proceso ideológico, los indígenas tienden a percibirse no solo como indígenas sino como “pobres”, y así en dos comunidades de Yucatán a finales de la década de 1970, observamos que población no nativa, pero también nativa decían no comer chaya -hierba que crece naturalmente por todos lados- pues es comida de pobres. Y algo similar señala Page respecto de indígenas de Chiapas, quienes dicen no comer verdura, pues es comida de pobres (comunicación personal en 2018). Es decir que los propios nativos se ven no solo como parte de un grupo étnico, sino como parte de un estrato social.

Los procesos de H/S/C no son coyunturales o esporádicos, sino que son procesos constantes. Y uno de esos procesos se da a través de la expansión biomédica sobre la población indígena rural y urbana que comienza y se desarrolla en el periodo colonial, pero que se va a acentuar en la segunda mitad del siglo XIX. Expansión que no solo se realiza a través de la presencia de personal de salud en las comunidades indígenas, sobre todo a partir de la década de 1920, por lo menos en Yucatán, sino también por la presencia de farmacias, por las venta de medicamentos en tiendas pequeñas, por la medicalización de los curadores locales, por la presencia de organizaciones no gubernamentales (ONG), de sacerdotes católicos y de pastores protestantes que promueven el uso de la biomedicina, cuestionando a la medicina tradicional. Cabe subrayar que, frecuentemente, las comunidades indígenas reciben atención médica de un tipo de personal de menor calificación, no solo por la ausencia de especialistas, sino porque gran parte de la atención se da a través de pasantes de Medicina. Y si bien, por ejemplo, en el caso del estado mexicano de Oaxaca, existen unidades médicas rurales ubicadas en zonas indígenas y rurales de Oaxaca, sin embargo “En Oaxaca el Sistema de Administración del Servicio Social para la salud penaliza a los pasantes que obtuvieron bajos promedios durante la carrera, ubicándolos en las localidades más retiradas de los centros urbanos”^19^. Pero pese a ello, la biomedicina se expande, porque si bien la población indígena tiene noción de que les envían “medicina de segunda”, sin embargo, demandan no solo médicos diplomados, sino hospitales. Esto es reconocido por varios de los principales analistas que, como Lozoya^20^, sostienen que, pese a su precariedad, la biomedicina seguirá expandiéndose, sin que la población indígena vuelva a usar solo medicina tradicional. 

Como señalamos previamente, en los pueblos originarios se acepta y se demanda cada vez más la biomedicina, dado que no se da la incompatibilidad entre ambos que han sostenido históricamente los analistas especializados en medicina tradicional, ya que como observamos tempranamente en nuestros estudios, por lo menos una parte de la población indígena utiliza tanto la medicina tradicional como la biomedicina como parte de su “carrera del enfermo”^21^. Esto ocurre no solo en México, sino a nivel latinoamericano, por lo que Freire^22^ sostiene que en ninguno de los grupos indígenas analizados en su libro:

…encontramos choques entre las concepciones indígenas de la salud, su tratamiento y exploraciones de la enfermedad y las de la biomedicina. Por el contrario, los pueblos indígenas incluso aquellos más alejados de la sociedad nacional geográfica y culturalmente, han incorporado buena parte de las terapias biomédicas a sus propias nociones de salud y enfermedad […] El derecho a un sistema de salud pública apropiado y de calidad es, de hecho, uno de los motores fundamentales del acercamiento de sus comunidades a la sociedad nacional y uno de los principales reclamos de sus organizaciones políticas de base.^22^

Posiblemente uno de los principales efectos, reconocidos por las poblaciones nativas, lo constituye la reducción de la mortalidad infantil, generada no solo por la atención, sino sobre todo por la prevención. Y así, por ejemplo, en Ocotepec (Chiapas), durante las décadas de 1970, 1980 y parte de la década de1990 hubo una gran mortandad por sarampión, tos ferina y cólera; más aún según sostiene un trabajador del Instituto Nacional Indigenista, en 1991 “prácticamente desaparecieron todos los niños debido a la epidemia de sarampión”^23^. Y si bien según Cortez sigue existiendo una alta mortalidad infantil en dicha comunidad, sin embargo, las tasas van descendiendo debido a las medidas preventivas y asistenciales biomédicas. No obstante, la expansión biomédica preventiva ha generado nuevos problemas, ya que si bien extirpó, por ejemplo, el paludismo que afectaba zonas indígenas, “nos dejó las consecuencias negativas del DDT” (comunicación personal con Pedro Yañez, 2023).

Pero no obstante estas y otras consecuencias negativas de las acciones preventivas, no cabe duda de que los nuevos problemas de salud que se han generado como la epidemia de covid-19 o el VIH-sida han tenido algunos de sus impactos más negativos en los pueblos originarios:

La situación que viven las personas indígenas con VIH en Ocosingo (Chiapas) y en Oaxaca son desalentadoras: sufren una mayor exposición a la infección y una mayor afectación que la población mestiza; una atención médica, un acceso al tratamiento y un seguimiento oportuno más inequitativo y, por ende, una mortalidad por sida mucho más alta. De igual forma, la transmisión vertical es mayor en las mujeres indígenas.^24^

Más aun, según Patricia Ponce, en las comunidades indígenas hay fuerte discriminación hacia las personas con VIH, por lo que quienes se han contagiado ocultan su problema (comunicación personal, 2016). Esta expansión biomédica se da, en gran medida, por la difusión directa e indirecta de saberes médicos como ya señalamos, que se van convirtiendo en hegemónicos; pero dicha hegemonía se desarrolla a través de la articulación que la población nativa hace entre la medicina tradicional y la biomedicina. Dicha articulación, si bien fue impulsada en forma pragmática por el sector salud, sin embargo, fue también desarrollada por la propia población indígena, a través de relacionar en los procesos de autoatención lo tradicional con lo biomédico. Esta actitud tiene que ver no solo con los recursos existentes a nivel comunitario, sino con una política del sector salud discontinua y pragmática, que la población necesita tomar en cuenta para atender sus enfermedades. Y así, por ejemplo, en 2005, se creó el Seguro Popular, que tuvo una gran penetración en la población indígena; pero en 2018 este programa se eliminó y el que lo sustituyó fue un fracaso, por lo que en zonas indígenas se volvió a la medicina tradicional, especialmente en el caso de las demandas de atención a las parteras y, sobre todo, a través de una autoatención, que incluía tanto lo que sabían de medicina tradicional como lo que habían aprendido de la biomedicina.

Desde finales de la década de 1970, el sector salud o el Instituto Nacional Indigenista trataron de impulsar un proceso de articulación, especialmente a través del reconocimiento que tanto la medicina tradicional como la biomedicina tenían de las plantas medicinales, pero todas las actividades propuestas hasta ahora han concluido en fracaso, en abandono o en mantenimiento letárgico. Y, como era de esperar, lo que se impuso es el uso biomédico de las plantas medicinales, pero a través de fármacos.

Como sabemos, la propuesta intercultural en salud planteó que las partes en relación -es decir, el personal de salud y los curadores tradicionales- tenían que tener un respeto mutuo, y no operar con discriminaciones ni exclusiones, lo que en la mayoría de los casos no ocurrió, dado que el personal de salud, por lo menos en México, no tiene interés en trabajar junto con curadores tradicionales, y menos en la misma institución. Y esto podemos observarlo en el caso de los hospitales y centros de salud interculturales impulsados por el Instituto Nacional Indigenista y por algunos gobiernos estatales, en los cuales se acentuaron en lugar de disminuir o borrarse las relaciones de hegemonía/subalternidad. Los centros mixtos o interculturales de atención incluyen, en una misma institución, el funcionamiento de la biomedicina y de la medicina tradicional, pero la manera de presentar a la población estos dos tipos servicios establecen de entrada relaciones de hegemonía/subalternidad en cuanto, por ejemplo, a los recursos que presentan los curadores tradicionales y los que presenta el personal de salud: en el caso de los primeros son mínimos comparados con los del personal biomédico, y esto es lo que experimenta la población que va a tratarse. Los hospitales mixtos reproducen las diferencias y distanciamientos entre curadores tradicionales y biomédicos, pero que en estos casos se da al interior de una misma institución.

En todas las instituciones en las que han trabajado con algún grado de articulación médicos alópatas y curadores tradicionales se genera una relación vertical, distanciada y discriminatoria, considerando a la medicina tradicional como vestigios superticiosos que es necesario erradicar. Y cuya máxima expresión está en el rechazo de los médicos a trabajar con brujos y con chamanes; lo que se puso de manifiesto en las instituciones mixtas impulsadas por funcionarios del Instituto Nacional Indigenista.

Y algo similar ha ocurrido con las organizaciones de curadores tradicionales impulsadas y financiadas inicialmente por el Instituto Nacional Indigenista, y que dio lugar a la formación de 102 organizaciones en diferentes estados, y a la constitución, en 1991, de una organización nacional: el Consejo Nacional de Medicina indígena Tradicional (CONAMIT), pero que en 2004 dejó de funcionar al igual que la mayoría de las organizaciones de médicos tradicionales, debido a la falta de financiamiento^25^. Subrayo que, en todas las experiencias interculturales en salud que conocemos en México, la medicina tradicional aparece subordinada a la biomedicina, pero además la casi totalidad de dichas experiencias concluyeron en fracasos o por lo menos en la exclusión de los curadores tradicionales por el sector salud, como ha sido el caso paradigmático de las parteras empíricas. Cabe recordar que dichas parteras empíricas fueron convocadas a partir de mediados de la década de 1970 para participar en el programa de extensión de cobertura y, especialmente, en el programa de planificación familiar, pero una vez cubiertas las necesidades del sector salud se excluyó a las parteras del trabajo de parto. En dichas experiencias no vemos capacidad de agencia por parte de los curadores tradicionales, ni el desarrollo de discursos y de prácticas que los coloque como una alternativa real a la biomedicina en términos no solo de sanadores, sino especialmente en términos contrahegemónicos.

Una parte de los intelectuales que apoyan estas experiencias han promovido, incluso a nivel constitucional, el reconocimiento de los usos y costumbres indígenas incluida la medicina tradicional. Y si bien en varios estados se han generado legalizaciones, estas no han hasta ahora favorecido el desarrollo de la medicina tradicional y menos su hegemonía o su papel contrahegemónico. Más aun, “se consolida un discurso de la interculturalidad en salud que no toca en ningún momento las causas estructurales de la injusticia social en las que se encuentra la mayoría de los pueblos indígenas”^26^.

Es decir que, aun en las experiencias, programas y propuestas generadas por intelectuales que apoyan a los pueblos originarios, observamos que se convalidan las relaciones de hegemonía/subalternidad, sin encontrar concreciones contrahegemónicas. Es interesante consignar que los intelectuales que impulsan los saberes indígenas y un “dialogo de saberes”, colocan la concreción de la hegemonía y la dominación en los sectores dominantes, pero no se preguntan por qué los sectores dominados no construyen propuestas y acciones contrahegemónicas. Y si se lo preguntan, señalan que no las construyen por culpa de los que hegemonizan y dominan, lo cual es obvio. Otros autores no entran a esta discusión, sino que consideran que más allá de la hegemonía/subalternidad, lo que importa para ellos es que la medicina tradicional permanezca, aun cuando descienda su uso cada vez más. Esto no niega el papel sanador y especialmente explicativo de los saberes tradicionales, sino que lo que plantea es que dichos saberes no solo son subalternos, sino que se van reduciendo en la vida de los actores subalternizados. Y lo que hay que explicar no es lo obvio -en este caso el papel de los sectores dominantes- sino que lo que hay que tratar de explicar es por qué no surgen procesos y acciones que buscan la hegemonía o por lo menos la contrahegemonía en el caso de los sectores subalternos y referidos a los procesos de salud-enfermedad-atención-prevención.

Algunas personas analistas proponen que, en los últimos años, especialmente en los estados de Oaxaca y de Chiapas -que junto con Yucatán son los estados con mayor población nativa-los pueblos originarios no demandan atención de los curadores tradicionales locales, sino que van a otras comunidades a tratarse con ellos. Esto ya lo habíamos documentado en dos comunidades de Yucatán a finales de la década de 1970; y según Güémez Pineda, esta tendencia, para pobladores de la comunidad de Pustunich, tiene que ver con que “no tiene caso ir a consultarle a una persona que conoce todo acerca de tu vida, y de tus problemas personales”^27^. Y este temor afianza aún más los usos biomédicos a nivel local.

Los procesos indican una constante tendencia a la hegemonía biomédica que, a finales de la década de 1970, en dos comunidades de Yucatán, se expresaba no solo en que la biomedicina era la medicina más valorada sobre todo para situaciones graves, sino que la más valorada era la medicina privada ejercida por especialistas comparada con la medicina del sector salud y, por supuesto, con la medicina tradicional^18^^,^^28^^,^^29^. Y algo similar ha encontrado Laura Montessi (comunicación personal, 2024), en comunidades de Oaxaca respecto de la diabetes mellitus 2, ya que los indígenas prefieren ir con médicos privados, pues consideran que estos no los van a amputar como si ocurre en las instituciones oficiales. Incluso, tanto en Yucatán como en Oaxaca, ocurre que médicos que trabajan simultáneamente en consultorios oficiales y en consultorio privado, son mejor valorados cuando ejercen como médicos privados. Es decir que es el costo financiero de la atención lo que determina la valoración de la atención médica, lo cual implica un proceso de hegemonización referido a las capacidades económicas diferenciales. 

Un aspecto importante para considerar es el que tiene que ver con una de las principales funciones de los curadores tradicionales, que es operar como control social a través del tratamiento de las enfermedades tradicionales. La emergencia de enfermedades tradicionales tiene que ver frecuentemente con la transgresión o quebrantamientos de reglas de comportamiento, lo que da lugar a que, en cada comunidad, haya un alto número de curadores tradicionales para actuar sobre las normas de comportamiento y las enfermedades. En 1984 el programa IMSS/COPLAMAR hizo una encuesta y encontró que en el área donde residía población indígena operaban 13.067 curadores tradicionales, lo que duplicaba el número de personal de salud que había en estas zonas, lo que verificamos en nuestro estudio sobre Ticul^18^^,^^28^^,^^29^. Por lo que una de las preguntas centrales refiere a quién resolverá ahora los conflictos que se pueden generar al interior de las familias o entre vecinos, y que si no se solucionan pueden llevar a agresiones que pueden concluir en la muerte. Y estas funciones no las puede cumplir el personal de salud.

## RELIGIÓN, ALCOHOLISMO Y OTRAS HEGEMONÍAS

Como señalamos al principio, la expansión de la biomedicina es parte de toda una serie de procesos no médicos que se potencian con la biomedicina para impulsar la hegemonía de los sectores sociales dominantes. Y dichos procesos se configuran en diferentes momentos, y algunos se mantienen hasta la actualidad. Por lo que a continuación voy a presentar información específica al respecto.

El catolicismo fue impulsado por los españoles a partir de reconocer en la población mesoamericana la existencia previa de creencias mágico-religiosas a las que había que reemplazar. Y esto se dio en forma constante entre los siglos XVI y XX, observando que desde la antropología tienden a ponderar la persistencia de las creencias religiosas prehispánicas en la población indígena actual, secundarizando el papel del catolicismo no solo respecto de los procesos religiosos, sino de los procesos de salud-enfermedad-atención-prevención. Y es debido a esto -y para evitar malos entendidos- que planteo observar en la situación actual cuál es el tipo de creencias que opera, y no entrar en una disputa por los orígenes, que se propone desde la antropología, dándole prioridad a lo prehispánico por razones básicamente ideológicas.

En la actualidad, además del catolicismo, asistimos a una expansión de religiones “protestantes”, especialmente en determinados pueblos originarios, lo que conduce a Baez-Jorge y Luppo^30^ a sostener que gran parte de los textos compilados por ellos sobre pueblos indios y cuestiones religiosas evidencian en la actualidad la hegemonía de las religiones católicas o protestantes en varios de dichos pueblos.

Ahora bien, esta disputa sobre los orígenes no se da solo en México, sino que opera en toda Latinoamérica. Y así, para el área andina y respecto del embarazo y parto, Platt sostiene que hay una oposición entre dos enfoques, uno que

…enfatiza el mestizaje de facto, biológico y cultural de todo habitante “indio”, en cuanto producto histórico de olas sucesivas de conquistas, cruces matrimoniales, colonialismo y transformación. Por el contrario, el otro enfoque enfatiza la persistencia indígena y su continuidad interna con sus predecesores precolombinos. Una posición busca desmitificar el indigenismo, tachándolo de “esencialista”; la otra defiende la “otredad” cultural y política de los indios andinos.^31^

Respecto de estas oposiciones necesitamos observar lo que ocurre con los procesos religiosos básicos; y en la actualidad no cabe duda -por ejemplo- que la principal imagen religiosa dominante en la sociedad mexicana es la Virgen de Guadalupe^5^^,^^32^, cuyo culto en la Nueva España fue impulsado especialmente a partir del siglo XVIII, y que según Brading,

…se ha dado gran importancia a las raíces prehispánicas de la participación indígena en el culto de Guadalupe, pero son escasos los indicios de la permanencia de una imagen prehispánica cualquiera de Tonantzin en el corazón o la mente indígena. En todo caso, desde sus inicios la devoción fue tan criolla como india y sin duda el clero criollo fue el responsable de su difusión a lo largo y ancho de los extensos territorios de la Nueva España.^33^

Por lo que considero que, lo que escribe periodísticamente José Agustín Ortiz Pinchetti, sintetiza el papel de la Virgen de Guadalupe:

Representa un símbolo de identidad para los mexicanos. Durante la época virreinal de la Nueva España, la devoción a la Virgen de Guadalupe unifico a indígenas, españoles, criollos y mulatos. En la independencia fue utilizada como símbolo por los insurgentes… La figura guadalupana es un fenómeno sociológico, cultural e histórico que por el hecho de ser mexicanos hemos sido parte de sus rituales. Me considero liberal, pero durante mi vida nunca me he desapegado de la Virgen de Guadalupe […] He pensado que si alguien pretende conocer al pueblo de México, sin duda lo que debe hacer es acudir un 12 de diciembre a los festejos de la Virgen del Tepeyac.^34^

Cabe recordar que el 12 de diciembre se genera en México la más numerosa peregrinación católica a nivel mundial, con procesiones de entre seis y doce millones de personas peregrinas, de las cuales una parte sustantiva la constituyen sujetos y grupos indígenas. Y esta virgen morena, no es cualquier virgen, sino que no solo es la patrona de América, sino que es la patrona de España: es la virgen frente a la cual los futuros conquistadores templaban sus armas antes de llegar a América.

Pero como señalé, pese a los procesos señalados, la tendencia de las interpretaciones antropológicas locales es colocar el peso de la religión que profesa la población indígena en el pasado prehispánico, considerando que los indígenas son más religiosos que los europeos, los cuales se caracterizan por su mayor racionalidad, lo que constituye una afirmación ideológica, ya que los indígenas se caracterizarían por

…una actitud inclusive pragmática y antidogmática común a gran parte de las sociedades “paganas”. Es probable que las concepciones y las prácticas religiosas de los amerindios hayan estado siempre sujetas a amplios márgenes de variación, adaptación y manipulación; de manera que las transformaciones recientes no constituyen una novedad radical, sino tan solo una relativa intensificación de procesos ya presentes, aunque en distinta forma y con otras motivaciones.^35^

Junto con la Virgen de Guadalupe, los usos del alcohol por parte de los pueblos originarios constituyen otro de los principales procesos que tienen que ver con la expansión colonizadora y la apropiación nativa, dado que el alcohol traído por los españoles se convertirá en parte nuclear de los rituales, ceremonias y fiestas realizadas por los indígenas mexicanos, incluidos los rituales de sanación^36^^,^^37^^,^^38^^,^^39^.

Más aún, el alcohol no solo es utilizado en rituales curativos, sino que el trabajo del curador es pagado con aguardiente. Y así, por ejemplo, entre la población indígena chol, los curadores tradicionales toman mucho alcohol e incluso realizan su trabajo sanador estando borrachos, Y además el alcohol puede tener que ver con la causalidad del mal de ojo, con la pérdida del alma, con la realización de “limpias” o con la cura y prevención respecto de los “aires”.

El alcohol es básico en las relaciones sociales, y puede generar disputas, pero también implica acercamientos. De allí que, por ejemplo, entre los indígenas triquis, cuando llega un visitante se le ofrece alcohol. Todo acuerdo entre sujetos o grupos se sella bebiendo juntos alcohol, dado que el consumo conjunto de alcohol implica que el acuerdo debe cumplirse necesariamente.

Debido a sus múltiples funciones y especialmente debido a la organización de fiestas comunitarias, alrededor del 50% de los gastos anuales de los grupos familiares son para la compra de bebidas alcohólicas. Por eso, y no solo entre los indígenas, se suele comprar y consumir alcohol adulterado cuyo precio es menor, pero que tiene consecuencias negativas para quienes lo beben, y que pueden concluir en la muerte.

La mayoría de estas acciones alcoholizadas no solo se dan en México, sino en la mayoría de los países latinoamericanos. Y es por estas y otras funciones que el consumo de alcohol no solo es vivido como proceso normal, sino como un proceso básico que es parte de la identidad cultural de los pueblos originarios. Incluso el alcohol no es vivido como problema o, si lo es, los sujetos y grupos siguen bebiendo aunque en ello se les vaya la vida. Y esto es así, porque en gran medida el alcohol es un integrador comunitario y no solo a nivel simbólico; ya que como señala El Guindi^40^, siguiendo a Selby, por ejemplo, los nativos zapotecos beben constantemente; siendo el alcohol el único mecanismo que ellos tienen cuando necesitan ayuda para resolver sus problemas, por lo que el consumo de alcohol constituye el único medio que tienen para adquirir seguridad social a nivel comunitario^40^.

Casi la totalidad de las descripciones antropológicas de los rituales que incluyen el uso del alcohol han sido realizadas por investigadores extranjeros. A continuación, sintetizaré dos de los principales estudios para ejemplificar estos procesos alcoholizados. El Guindi realizó su investigación sobre una comunidad indígena oaxaqueña y observó que los rituales alcoholizados se dan con mayor frecuencia en los bautismos, en los casamientos y, sobre todo, en los funerales. En las tres ocasiones se bebe constantemente hasta la embriaguez. En el caso de los funerales, uno de los más importantes es el velorio del angelito, que se realiza sobre todo respecto de muertes de niños de menos de un año. En todos los pasos ceremoniales de este velorio se bebe, sobre todo, mezcal. De tal manera que, a la gente que llega al funeral, se le da mezcal; cuando se viste al angelito se lo bendice con mezcal; cuando se baila, se toma mezcal; en la procesión hacia el cementerio, se sigue bebiendo; y, al llegar al entierro, el padrino del niño muerto y los familiares están al lado de la fosa bebiendo mezcal. El funeral puede durar varios días y uno de ellos recibe el nombre de “día de la botella”, que implica que sobre todo los familiares beben durante todo el día mezcal bendecido. En el caso de los funerales de los adultos también se bebe continuamente alcohol, y hay dos “días de la botella”^40^.

A su vez, Fabrega y Silver^41^ describieron el consumo de alcohol en Zinacantan (Chiapas), donde el aguardiente (*posch*) constituye uno de los principales medios de interacción de los hombres entre sí y de los hombres con los dioses. Una de las principales características del alcohol es su presencia en la curación chamánica, en la que los participantes, incluido el chaman, beben alcohol. El chaman recibe ciertos regalos por sus servicios sanativos, siendo el alcohol el principal regalo a través de siete botellas de aguardiente que debe consumir, ya que el alcohol no debe ser acumulado.

Ahora bien, estos rituales alcoholizados que favorecen la integración comunitaria contrastan con las consecuencias negativas de los usos del alcohol. Ya hemos señalado que el escape del alma puede estar relacionado con borracheras, al igual que el espanto; así como el uso del alcohol aparece relacionado con las brujerías, ya que todo alcoholismo puede ser atribuido a estas en diferentes contextos mexicanos. Pero es la violencia, en general, y la violencia intrafamiliar, en particular, donde observamos las consecuencias más graves de los usos del alcohol. Más aún, gran parte de los homicidios aparecen relacionados con el consumo de alcohol^42^.

Pero ocurre que, por ejemplo, si bien en las fiestas comunitarias se generan diferentes problemas debidos al consumo de alcohol, las autoridades locales tienden a minimizar sus acciones represivas contra los ebrios que generaron las agresiones. Estos procesos conducen a que el alcoholismo se constituya en una de las principales causas de muerte en pueblos originarios en forma directa e indirecta, y así en los municipios con 70% o más de población indígena, la cirrosis hepática es la primera causa de muerte en varones, mientras el homicidio es la segunda causa y el uso de alcohol la séptima; mientras, en el caso de las mujeres, la cirrosis es la segunda causa de muerte y el homicidios la sexta^43^.

En general, los hombres no cuestionan el consumo de alcohol, aunque sí sus mujeres, dado que atribuyen al alcohol gran parte de las agresiones que los varones ejercen contra ellas. Los hombres agreden a sus mujeres con cualquier pretexto y, especialmente por celos, pero las castigan verbal y físicamente cuando están borrachos. Lo que surge de las etnografías que tratan sobre alcoholismo, es que todo tipo de violencias pueden estar relacionada con los usos del alcohol; pero, además, la mayoría de las agresiones son contra familiares, vecinos o conocidos de la comunidad; es decir, contra personas cercanas.

Como señalé, el alcohol tiene que ver con las relaciones entre las personas y con las relaciones con los dioses; y no hay que olvidar que, durante la misa, el sacerdote bebe vino que representa la sangre de Cristo y los fieles que comulgan comen la hostia, que simboliza el cuerpo de Cristo. El alcohol aparece no como causa de una enfermedad llamada alcoholismo sino que, por el contrario, significa “calor vital”, salud, medicina, y por eso el consumo de alcohol aparece no solo normalizado, sino protegido por las familias y por las autoridades comunitarias. Esto es refrendado tanto por la medicina tradicional como por la biomedicina, dado que el alcohol de 96° es una sustancia usada para curar, pero también para embriagar. El alcohol posibilita que el ebrio diga la “verdad” y, por eso, y por otras razones, el sujeto cuando esté borracho debe ser protegido. Pero, al mismo tiempo, el alcohol es parte de homicidios y de violaciones sexuales. El alcohol es, además, una importante causa de accidentes, ya que los sujetos embriagados se caen, se desbarrancan, se despeñan, corren el peligro de ahogarse, y son estos accidentes los que pueden ser reformulados como pérdida del alma o como susto.

Todos estos procesos, que son parte sustantiva de la vida indígena, están relacionados con un producto que trajeron los españoles, y que una parte de los mismos indígenas van a aprender a elaborar. El alcohol es una de las sustancias que más han transformado determinados aspectos de la vida cotidiana indígena, actuando como una sustancia que conduce a una suerte de control comunitario, que posibilita que la mayoría de las agresiones se realicen no contra quienes los marginan, los explotan, los racializan, sino contra sus pares culturales. 

Estos usos del alcohol por parte de los pueblos originarios generan simultáneamente cohesión y violencias internas^44^; y no constituyen solo un proceso planificado por los colonizadores y luego por la sociedad criolla, sino que surgen del propio funcionamiento cotidiano de los indígenas, favoreciendo las relaciones de hegemonía/subalternidad dominante, pero no dando lugar a procesos contrahegemónicos. Entendiendo que el alcohol no es quien genera las consecuencias negativas o positivas, sino que son los usos del alcohol por sujetos y grupos los que las generan. Por lo que el problema no está en el alcohol, sino en quienes lo usan; y entre quienes lo usan puede aparecer como mecanismo de resistencia, pero no de contrahegemonía.

## CONTRAHEGEMONÍAS

A lo largo del texto hemos analizado varias temáticas en las que observamos procesos de hegemonización generados por los sectores sociales dominantes, así como la escasez de procesos contrahegemónicos, por lo que ahora voy a tratar de puntualizar la existencia o no de estos últimos procesos. El primer punto a reconocer es que hay toda una serie de propuestas que subrayan las diferencias de los pueblos originarios y de la medicina tradicional, y que consideran que dichos pueblos constituyen una alternativa a las sociedades capitalistas y, en particular, al neoliberalismo, sobre todo cuando logran un empoderamiento comunitario según algunos, o étnico, según otros. Estas propuestas cuestionan a nivel académico e ideológico las visiones e interpretaciones que la historia y la antropología han dado de los pueblos originarios, y sostienen además que se necesita crear una conciencia étnica que unifique e impulse acciones, lo cual es cada vez más necesario debido a los procesos de aculturación/hibridación existentes. Pero lo que más se requiere es de-colonizar los saberes impuestos por los sectores dominantes e impulsar la autonomía territorial.

Y estos procesos, según los neoindianistas, deben ser realizados por los propios indígenas; ya que los no indígenas

…no pueden participar de un proyecto de descolonización que debe ser emprendido por miembros de las sociedades indígenas, sobre todo, cuando de lo que se trata es de revertir una lógica de producción de conocimiento, cuestión en la que los propios indígenas deben ser protagonistas.^45^

Esto implica el desarrollo de una epistemología indígena, según unos, o de una epistemología del sur, según otros, como parte del proceso de descolonización; y denuncian que la ciencia es parte de la colonización, ya que maneja una epistemología eurocéntrica. Por lo tanto, se necesita desarrollar una epistemología desde la periferia, desde el lugar de vida de los pueblos indígenas^46^. Y junto con ella se requiere desarrollar las concepciones que revalorizan la cultura de los pueblos indígenas, para evidenciar que no es inferior a la de aquellos que los colonizaron y los siguen dominando. Se busca poner de manifiesto la diferencia y la identidad cultural de dichos pueblos, colocando todo el peso en reivindicar la etnicidad.

Y estas propuestas suponen una reconstrucción mitificada del pasado que las fundamenta; reconstrucción, que más allá de su verdad, busca legitimar los objetivos de descolonización en relación con un “otro”:

…creo que el factor clave que lleva a un grupo a mantener su identidad o a enfatizar sus diferencias, es la dominación que ejercen los miembros de un grupo externo […] La afiliación étnica prevé un mundo social alternativo en que la gente pueda tener éxito según sus propios criterios y defenderse de la violencia del grupo dominante.^47^

De tal manera que la mitificación del pasado, que el esencialismo que manejan los intelectuales neoindios, constituye una estrategia descolonizadora, que puede o no coincidir con el pasado, lo que pasa a ser secundario, por lo menos en términos ideológicos. Consideran que, en la actualidad, la identidad indígena es una construcción con objetivos políticos y sociales; por ello, es decisivo construir un imaginario a partir de la diferenciación con el otro que nos domina, para posibilitar la cohesión y la movilización. Cabe subrayar que otras tendencias indianistas proponen acceder realmente al pasado, cuestionan que eso constituya una invención, y se preguntan cómo nos reconectamos con la cultura ancestral. Y si bien estas propuestas ya estaban formuladas en la década de 1940 y 1950 por el relativismo cultural, por Fanon, o por el grupo Socialismo o Barbarie, ahora aparecen resignificadas y referidas a los grupos nativos americanos por intelectuales indígenas y por decoloniales en su mayoría “blancos”.

Frente a las concepciones que sostienen que las poblaciones indígenas se ven obligadas estructuralmente a entrar a un mercado de trabajo capitalista que implica fuertes modificaciones en su subjetividad social, se recuerda que por lo menos una parte de los pueblos nativos producen para el mercado, pero también producen para el autoconsumo como proceso no solo económico, sino también cultural. Otro de los cuestionamientos más frecuentes a estas propuestas, es que están escritas en español o portugués, y que utilizan concepciones teóricas y conceptos formulados por las ciencias occidentales, especialmente, europeas.

Estas tendencias buscan la “verdad” en el pasado, mitificado o no, de los pueblos nativos como instrumento que posibilite la cohesión y la movilización. En principio, esto plantea, por lo menos, dos interrogantes: primero, ¿estas propuestas decoloniales y neoindias son creadas e impulsadas exclusivamente por intelectuales indígenas y no indígenas, o la población indígena interviene también en la formulación de estas propuestas y las lleva a cabo? Y, segundo, ¿la población nativa se reconoce en la construcción de ese pasado que supuestamente les pertenece? Dado lo que estamos observando y leyendo, en México, son propuestas básicamente intelectuales, con escaso uso por parte de la población indígena.

Por otra parte, si observamos la construcción de estas propuestas desde los procesos de salud-enfermedad-atención-prevención, queda claro que los intelectuales excluyen toda una serie de procesos básicos para construir un pasado mitificado, de tal manera que no describen ni analizan las altas tasas de mortalidad ni la reducida esperanza de vida de los pueblos nativos en el pasado y en el presente, no asumen la falta de eficacia de la medicina tradicional respecto de las enfermedades alopáticas que, en la actualidad, constituyen las principales causas de mortalidad en la mayoría de los pueblos originarios. Simultáneamente, excluyen analizar la penetración no solo del alcohol, sino de los alimentos chatarra en la cultura alimenticia de los indígenas mexicanos.

Y así observamos que el sobrepeso y la obesidad se están dando a nivel nacional en los sectores más pobres y, especialmente, en las poblaciones indígenas:

...precisamente en estas regiones indígenas es donde se observan las prácticas de mercadeo más agresivas y no reglamentadas para promocionar el consumo de bebidas azucaradas, incluyendo una reducción generalizada del 35% del precio de las mismas, abundante publicidad en español y en lenguas indígenas, anuncios gigantes que asocian el consumo de refrescos con símbolos tradicionales de prestigio, así como numerosos puestos de venta dentro y en los alrededores de las escuelas. Según sus datos, en la región indígena de Tenejapa (Altos de Chiapas) existen 165 puntos de venta de refrescos […] es decir que hay un punto de venta cada 257,5 metros.^48^

Y al respecto me interesa subrayar que esta alimentación afecta el cuerpo de los nativos no solo en términos de obesidad y sobrepeso, sino en términos de amputaciones generada por diabetes mellitus 2 no tratadas. Es decir, los nuevos productos afectan profundamente el cuerpo físico y cultural de la población indígena; tan profundamente que se convierte en una de las principales causas de mortalidad. Y si bien la población nativa utiliza la medicina tradicional para enfrentar estos problemas y no solo la medicina alopática, sabe que su medicina no cura este padecimiento, así como también sabe que quienes los van a amputar son biomédicos y no curadores tradicionales.

Así como también esta reconstrucción mitificada del pasado y del presente excluye la situación de inferiorización sistemática de la mujer indígena en todos los planos de la realidad; inferiorización que está basada en los valores culturales nucleares de los pueblos indígenas. Y cuando se proponen como alternativa al capitalismo no he observado hasta ahora qué tipo o tipos de sistemas de salud proponen, y digo tipos porque muchos proponen su alternativa al capitalismo a partir de las comunidades.

Guillermo Bonfil Batalla^49^ es uno de los antropólogos no indígenas que más ha elaborado concepciones proindígenas, considerando que la cultura indígena no es una parte de la cultura dominante en México, sino que consiste en una totalidad en sí. La cultura indígena no es un subnivel subordinado, sino que necesitamos tratarla como una entidad diferente en todos los campos de la realidad. Dado que esa cultura mantiene los aspectos que la identifican y la diferencian de una cultura occidentalizante. Y por más que Bonfil nos habla de cultura popular al referirse a la cultura indígena, su propuesta tampoco ha impulsado organizaciones y movilizaciones populares. Considero que las culturas indígenas mexicanas sí son parte de la sociedad mexicana, cuya máxima expresión lo constituye el continuo mestizaje, que tiene como una de sus partes a los hombres y las mujeres indígenas que, entre otras cosas, como ya señalé, constituyen el principal reservorio de mano de obra para el desarrollo capitalista de México, y en parte de los EEUU.

Bonfil y los grupos de analistas proindígenas parecen no reconocer que están disminuyendo y hasta desapareciendo los diferentes tipos de curadores tradicionales, cuyas acciones aparecían como contrahegemónicas, dado que seguían manteniendo las formas ancestrales de diagnóstico y tratamiento; y que, algunos de ellos, como en el caso de los chamanes y brujos, tenían que ver con la detección y tratamiento de padecimientos mentales^50^. Más aun, esta disminución corre paralela a que parte de los curadores tradicionales tengan demanda por parte de turistas, generándose un proceso de mercantilización en el cual la medicina tradicional aparece como espectáculo, como es el caso emblemático de las reuniones de los brujos de Catemaco (estado de Veracruz), que periódicamente se citan unos 50 brujos y llegan miles de turistas a hacerse limpias, a comprar amuletos, y a deshacer hechizos. O lo ocurrido en Yucatán, donde el gobierno provincial envió cuatro “sacerdotes” mayas a la Feria Internacional de Turismo de Berlín (Alemania). El caso más paradigmático son los usos de los hongos mágicos por Doña Sabina, que implicó una corriente turística internacional hacia la comunidad oaxaqueña donde ejercía dicha curadora (Piña 2021). Y este proceso no se reduce a México, ya que en Venezuela hay un extenso proceso de mercantilización en el caso de los chamanes.

Paradojalmente, los intentos del gobierno de Chávez por legitimar e institucionalizar el chamanismo tradicional en años recientes, creando centros de curación y tratamiento chamánico. Y ofreciendo salarios a los chamanes que trabajaban ahí, parece destinado a acelerar este proceso de profesionalización capitalista más aún.^51^

Mercantilización que opera también en el caso de los bailes, de las artesanías y, especialmente, en la producción textil tradicional. En este último caso se generó en los últimos años un proceso reiterado, dado que importantes casas de modas a nivel mundial utilizaron diseños textiles tradicionales mexicanos, lo que dio lugar a que artesanas indígenas denunciaran esta situación, y solicitaron se diera denominación de origen a sus diseños^52^. Por otra parte, organizaciones de artesanos mexicanos se quejan de que el mercado turístico de México está invadido por artesanías “mexicanas” producidas en China, lo que reduce cada vez más sus posibilidades de venta de las “verdaderas” artesanías.

La mercantilización necesita ser articulada con toda una serie de procesos, de los cuales solo recordamos algunos; y así observamos que cada vez más la población indígena depende de un mercado capitalista de trabajo, que vive en un constante proceso de emigración rural/urbana y rural/rural en busca de trabajo, que cada vez más los nativos viven en medios urbanos, que en sus comunidades disminuyen las fiestas y sistemas de cargos, así como también se reduce la existencia de las más importantes enfermedades tradicionales^53^^,^^54^. 

Pensando en términos contrahegemónicos, considero que los intelectuales proindígenas han desarrollado algunas propuestas que no sabemos todavía cuáles serán sus consecuencias; tal vez, las más interesantes son las que señalan al comunalismo como eje de todo desarrollo indígena; las que proponen la autonomía en todos los campos de la realidad, que se expresaría a través de los caracoles zapatistas; así como el concepto de “buen vivir”, que es el que ha tenido más desarrollo en Latinoamérica, aunque no demasiado en México. Si bien existen antecedentes, esta última propuesta comienza a desarrollarse desde la década de 1970, pero pasa a tener visibilidad a finales de la década de 1990 y, sobre todo, durante el 2000. Se desarrolla sobre todo en Ecuador y Bolivia, y escasamente en México; y se desarrolla cuando se desacelera el papel de los movimientos étnicos. Este concepto surge en oposición al “mal vivir” occidental y capitalista, y se caracterizaría por la reciprocidad, ayuda mutua, solidaridad, saber compartir, armonía entre las personas, equilibrio físico y espiritual, bienestar individual y colectivo; centralidad de la comunidad en la vida colectiva; el respeto y la utilización de los saberes ancestrales; la espiritualidad en las relaciones con todos los seres vivos; el equilibrio con la naturaleza para satisfacer las necesidades; la erradicación de la pobreza, la distribución equitativa de bienes y, sobre todo, la relación con la “madre tierra”^55^^,^^56^^,^^57^.

Mientras una parte de los neoindios proponen el buen vivir en forma exclusiva para los pueblos originarios, otros lo remiten a toda sociedad ya que, según Briggs, esta propuesta

…va más allá de la incorporación de cosmovisiones indígenas en modelos abstractos o la búsqueda de procesos paralelos en lo que se pinta como cosmovisiones o modelos autónomos. En vez de ofrecer un modelo “multicultural” de universos separados, desarrollamos una práctica intercultural critica.^58^^)^

Para proponer estas características que serían propias de las culturas indígenas, los intelectuales indígenas y los postmodernistas necesitan excluir toda una serie de procesos que cuestionan dicho “buen vivir”, y que en gran medida ya hemos citado, y me refiero a las altas tasas de mortalidad y a la reducida esperanza de vida, así como a la mortalidad por causas fácilmente evitables; incluyendo la existencia de conflictos entre sujetos y grupos cercanos, así como la situación de inferioridad y cosificación de la mujer que supone, entre otras posibilidades, la compra de la mujer para establecer casamientos entre adultos y casi niñas, lo que constituye un “buen vivir” que -no olvidemos- implica la continua migración masiva desde sus tierras de origen hacia los territorios del “mal vivir”.

Por supuesto que los que proponen el “buen vivir” saben de estos problemas, pero lo que buscan es construir concepciones ideológicas que posibiliten la cohesión y la movilización para lograr sus objetivos de autonomía territorial y cultural. Por lo tanto, la mitificación del pasado, las exclusiones de los problemas y el buen vivir constituyen construcciones para poder transaccionar con los sectores dominantes y lograr su autonomía cultural y territorial. Y la cuestión radica en si están o no consiguiendo dicho objetivo.

Por lo tanto, el buen vivir es un concepto básicamente ideológico que puede ser articulado con propuestas políticas desarrolladas por movimientos étnicos, así como por sus intelectuales. Pero también puede ser apropiado por importantes representantes de la medicina social critica, como es el caso de Breilh, para quien

...el buen vivir es una idea potente, una de esas ideas que son indispensables en épocas de inconformidad y transformación social para orientar la lucha de las colectividades […] La noción de buen vivir tiene asidero en la cosmovisión indígena […] pero es también cierto que la noción de buen vivir no existe únicamente en la cosmovisión indígena, puesto que nociones similares están presentes en otras formaciones culturales, y está presente desde la década de 1970 en el movimiento de medicina social de América Latina.^59^

Para Breilh, al igual que para los movimientos indígenas, el buen vivir se opone al malvivir capitalista, y plantea la necesidad de desarrollar una estrategia unificada. Y también, al igual que los intelectuales proindígenas y los postmodernistas, excluye todos los procesos negativos que refieren a un mal vivir en las sociedades indígenas. Más aun, un intelectual proindígena mexicano sostiene que el buen vivir nativo se caracteriza no solo por constituir la alternativa actual al capitalismo, sino porque gozan de un buen vivir más feliz que el resto de las sociedades^60^.

Por supuesto, no ignoramos y menos negamos los procesos que en Ecuador y en Bolivia resituaron a los pueblos originarios como parte central del escenario político, pero en la medida que asumamos que esto ha conducido a conformarlos como organizaciones políticas que necesitan entrar a juegos de poderes, que van secundarizando sus propuestas autonómicas. Esto se verifica en el caso de la medicina tradicional y de la biomedicina, ya que si vemos en el único país donde sectores indígenas llegaron al poder, y me refiero a Bolivia, el gobierno impulsó exclusivamente la biomedicina, invirtiendo en ella, mientras nombró una subsecretaria de medicina tradicional, pero sin recursos humanos ni financieros.

Ahora bien, no negamos que los pueblos indígenas para sobrevivir generen resistencias, pero la cuestión es si esas resistencias les sirven para “aguantar” la situación en la que viven o si también implican el desarrollo de contrahegemonías que realmente estén operando. Para Scott^61^^,^^62^, por ejemplo, la migración o las violencias e incluso el crimen organizado pueden ser resistencias, pero la cuestión es ver qué sentido tienen estos actos y qué generan en los actores hegemónicos y en los subalternos. Y todo indica que, por lo menos, respecto de los procesos de salud-enfermedad-atención-prevención, se pueden generar resistencias como el rechazo a la mala y racista atención médica o se pueden proponer e impulsar concepciones contrahegemónicas como las del buen vivir, pero lo que observamos es que en el primer caso sigue expandiéndose la biomedicina, y reduciéndose la medicina tradicional; mientras que, en el segundo, solo queda, en el caso de México, como proyecto intelectual.
